# Molecular Epidemiology and Antibiotic Resistance of Sheep-Derived *Mannheimia haemolytica* in Northwestern China

**DOI:** 10.3390/ani15233492

**Published:** 2025-12-04

**Authors:** Chenxiao Wang, Leina Dou, Juan Wang, Dongyang Ye, Zengqi Yang

**Affiliations:** 1 Department of Preventive Veterinary Medicine, College of Veterinary Medicine, Northwest A&F University, Yangling 712100, China; chenxiaowang@nwafu.edu.cn (C.W.); douleina1717@nwsuaf.edu.cn (L.D.); juan.wang@nwafu.edu.cn (J.W.); yedongyang@nwafu.edu.cn (D.Y.); 2 Key Laboratory for Prevention and Control of Major Ruminant Diseases, Ministry of Agriculture and Rural Affairs, Yangling 712100, China

**Keywords:** ovine-derived *Mannheimia haemolytica*, pathogenicity, drug resistance, virulence genes

## Abstract

This study comprehensively explored the epidemiology, virulence, antibiotic resistance, and genomic features of ovine-derived *Mannheimia haemolytica* across three provinces in Northwest China (Gansu, Ningxia, and Shaanxi). Lung tissue samples were collected postmortem from sheep with respiratory disease (nine per province), cultured under sterile conditions, and screened by morphological, Gram staining, PCR, and NGS methods. Nine *M. haemolytica* strains were successfully isolated (isolation rate 33.33%) and identified as serotypes A1, A2, and A6, with Shaanxi exhibiting a significantly higher isolation rate of 66.67%. Genomic analysis revealed that each isolate each harbored over 100 virulence genes and displayed notable genetic diversity. In murine assays, intraperitoneal inoculation in mice caused severe breathing problems and death within 24 h; necropsy revealed lung congestion, splenic necrosis foci, and hepatic congestion. Antibiotic susceptibility testing showed sensitivity to ciprofloxacin, azithromycin, gentamicin, and levofloxacin, while resistance was observed for tiamulin and enrofloxacin. These findings indicate a high prevalence and strong pathogenicity of ovine *M. haemolytica* in Shaanxi, underscore the presence of key resistance traits, and provide a genomic and pathogenic reference to guide region-specific surveillance, therapeutic decisions, and vaccine-oriented control in small ruminant herds. Importantly, these results highlight the need for region-specific monitoring and judicious antibiotic use, which are essential to forestall the spread of resistant strains and to ensure sustainable disease management strategies.

## Simple Summary

Respiratory disease in sheep caused by the bacterium *Mannheimia haemolytica* poses a serious threat to small-ruminant farming in northwest China. In this study, we collected lung tissue from sheep that died of suspected respiratory infection in three provinces, isolated nine bacterial strains, and conducted whole-genome sequencing, virulence gene profiling and antibiotic resistance testing. We found that most strains carried key virulence genes and showed high resistance to specific antibiotics, and importantly, strains from one province exhibited both higher virulence and stronger resistance. These findings will help guide region-specific monitoring, inform prudent antibiotic use and support the development of targeted vaccines and control strategies for sheep flocks in this region.

## 1. Introduction

*M. haemolytica* is a Gram-negative bacterium of the family Pasteurellaceae, commonly found in ruminants such as sheep, goats, and cattle [1]. It is a significant pathogen in the bovine respiratory disease complex (BRDC) and is widely distributed across the globe, particularly in regions with developed livestock industries [2,3]. As an important pathogen, *M. haemolytica* plays a crucial role in the development of pneumonia and septicemia in livestock, causing substantial economic losses—estimated in the U.S. beef industry at over USD 1 billion annually [4]. Given the severe threat posed by *M. haemolytica* to the sheep industry, a comprehensive understanding of its epidemiology, pathogenic mechanisms, and antimicrobial resistance profiles is of paramount importance for devising effective control and prevention strategies [5].

In China, the epidemiological patterns of *M. haemolytica* exhibit marked diversity due to regional differences, particularly across various climate zones with complex ecological environments. Extreme natural conditions not only affect the health status of livestock in local pastures but also have profound impacts on the transmission and evolution of pathogens [6]. For instance, in the northwest provinces, there are notable variations in climate and geography, as well as diverse husbandry practices among herders, leading to unique genetic diversity and resistance characteristics of *M. haemolytica* strains in these regions [7].Therefore, delineating the distribution of *M. haemolytica* in the northwest region is highly informative for interpreting this epidemiologic phenomenon.

The pathogenicity of *M. haemolytica* is closely associated with the virulence factors it harbors, particularly the expression of virulence genes, which markedly influences the severity of host infection. Known virulence factors of *M. haemolytica* include outer membrane proteins, extracellular enzymes, and hemolysins [8,9,10]. *lktA*, its main virulence determinant, belongs to the RTX toxin family and can specifically recognize and lyse host neutrophils and macrophages, leading to severe inflammatory responses and pulmonary tissue necrosis [11]. *OmpA* is not only involved in bacterial adhesion and invasion but also resists complement-mediated killing, enhancing its colonization ability in the host respiratory tract [12]. *FhaB*, as an important adhesin, mediates the binding of bacteria to host epithelial cells and participates in immune regulation, promoting the establishment of infection [13]. The *tonB* protein in the iron uptake system drives the outer membrane receptors to take up iron ions from host iron-binding proteins, supporting bacterial growth and survival in iron-limited environments and is crucial for in vivo virulence [14]. In addition, *sodA* helps bacteria resist oxidative stress by clearing superoxide anions produced by host immune cells, maintaining their survival ability in inflammatory environments [15]. These factors facilitate the pathogen’s evasion of the host immune system, disrupt host cells, and cause severe tissue damage. Furthermore, the presence and expression patterns of specific virulence genes show a significant correlation with the pathogenicity of strains isolated from different regions [16]. The detection and analysis of virulence genes contribute to predicting the pathogenic potential of *M. haemolytica*, thereby providing a basis for clinical treatment and vaccine development.

In recent years, the issue of antibiotic resistance in *M. haemolytica* has become increasingly serious due to the wide spreading use of antibiotics in livestock farms. The misuse of antibiotics has led to the gradual development of resistance to several commonly used drugs, particularly β-lactams, aminoglycosides, and tetracyclines [17]. The rise in resistance not only complicates the treatment of infections but also poses threats to animal health and food safety. Detecting resistance genes in *M. haemolytica* can help assess the resistance profiles of strains from different regions, providing a scientific basis for formulating rational antibiotic usage strategies.

Next-generation sequencing (NGS) technology not only accurately reveals the structural characteristics of *M. haemolytica* genomes but also aids in a deeper understanding of its pathogenic mechanisms, gene mutations, and the distribution patterns of resistance genes [18]. Current research indicated that the virulence factors and resistance genes of *M. haemolytica* are closely associated with its pathogenicity; thus, detecting these genes is instrumental in better predicting the bacterium’s virulence and its response to antibiotic treatment [19].

In summary, the study aimed to conduct whole-genome NGS analysis of *M. haemolytica* strains isolated from three provinces in the northwest region, exploring their epidemiological characteristics and the distribution of resistance and virulence genes. Through an in-depth analysis of the sequencing data, combined with regional epidemiological survey results, this research not only offers new insights into the pathogenicity of *M. haemolytica* in the region but also provides scientific evidence for controlling and preventing the spread of this pathogen, ultimately aiming to furnish comprehensive information for the development of effective control strategies.

## 2. Materials and Methods

### 2.1. Sample Collection

The study was conducted in three northwestern provinces of China (Gansu, Ningxia, and Shaanxi), where lung tissue specimens were collected from sheep that died with signs of respiratory infection, obtaining nine samples per province. Only domestic sheep of local breeds, reported by farm records to have displayed clinical respiratory signs immediately prior to death and free from concurrent major disorders were included. Strict aseptic procedures were maintained throughout sampling. In total, 27 lung tissue samples from diseased animals—each showing clinical respiratory symptoms or pulmonary lesions at necropsy—were gathered. According to farm records, animals’ ages ranged approximately from 5 months to 9 months, and none received documented antibiotic treatment prior to death. Specimens were promptly transported on cold chain to the laboratory and stored at −20 °C.

### 2.2. Pathogen Isolation and Identification

Trypsin soybean agar (TSA) (Oxoid, Basingstoke, Hampshire, UK) was selected as the solid culture medium. Sterile saline was concurrently plated on TSA as a negative culture control. Under aseptic conditions, the collected sheep lung tissues were inoculated onto the surface of the TSA plates and incubated in a thermostatic incubator (Thermo Fisher Scientific, Waltham, MA, USA) at 37 °C for 24–48 h. After 24–48 h, the growth of bacterial colonies on the medium was assessed by visual inspection and microscopic examination (Olympus, Tokyo, Japan). Isolated colonies of *M. haemolytica* typically exhibited circular morphology, measuring 1–2 mm in diameter, with a smooth surface and well-defined edges, accompanied by the formation of β-hemolytic zones. The colony morphology and hemolytic characteristics were observed to aid in preliminary identification. Subsequently, individual typical colonies from the blood agar plates were selected for Gram staining. *M. haemolytica* is characterized as a Gram-negative short rod, appearing as pink short rods under the microscope, arranged either randomly or in chains. Additionally, another portion of the colonies was inoculated into Martin broth containing 5% horse serum (Sigma-Aldrich, St. Louis, MO, USA) for at least three serial passages, until the staining results confirmed the presence of only the target bacterium. This process ensured the isolation and purification of the bacteria, after which the purified bacterial suspension was preserved.

### 2.3. DNA Extraction and 16S rRNA Gene Amplification

Genomic DNA was extracted from the isolated strains following these specific steps: First, a single fresh colony was selected and suspended in 1 mL of sterile saline solution, thoroughly mixed. DNA extraction was then performed using the bacterial genomic DNA extraction kit (OMEGA, Norcross, GA, USA), adhering strictly to the manufacturer’s instructions. The process included cell lysis, protein removal, and DNA purification, ultimately yielding high-purity genomic DNA. The concentration and purity of the extracted DNA were assessed using a NanoDrop spectrophotometer (Thermo Fisher Scientific, Waltham, MA, USA), ensuring an OD_260/280_ ratio between 1.8 and 2.0 to meet the requirements for subsequent experiments.

The 16S rRNA gene was amplified using polymerase chain reaction (PCR) technology. The amplification reaction system comprised 25 μL, including 2× PCR Master Mix (Thermo Fisher Scientific, Waltham, MA, USA), 10 pmol/L of universal upstream primer (27 F) sequence: 5′-AGAGTTTGATCCTGGCTCAG-3′ and downstream primer (1492 R) sequence: 5′-TACGGCTACCTTGTTACGACTT-3′, template DNA (10 ng), and sterile water. The PCR program was set as follows: initial denaturation at 94 °C for 5 min; 35 cycles of denaturation at 94 °C for 30 s, annealing at 55 °C for 30 s, and extension at 72 °C for 1 min; followed by a final extension at 72 °C for 10 min. After amplification, a portion of the PCR products was analyzed using 1.0% agarose gel electrophoresis. The electrophoresis buffer used was 1× TAE (Solarbio, Beijing, China), and the gel was run at 120 V for 30 min. DNA bands were stained with nucleic acid dye (DiNing, Beijing, China). The amplified bands were observed using a UV gel imaging system (Clinx, Shanghai, China) to confirm the quality and specificity of the amplification, remaining products were subjected to gene sequencing (Tsingke Biotech, Beijing, China).

### 2.4. Serotype Identification

According to related reports, *M. haemolytica* types A2 and A6 are commonly isolated from sheep [20,21]. A multiplex PCR assay, adapted from previously established protocols (Table 1), was employed to determine the capsular serotypes of *M. haemolytica* [22]. Using serotype-specific primer sets targeting A1, A2, and A6, the assay was optimized to function under a unified annealing temperature of 55 °C. Amplification products were resolved by agarose gel electrophoresis, with serotype assignment based on expected amplicon lengths. This validated multiplex approach enables simultaneous, accurate, and cost-effective differentiation among the three most prevalent and clinically significant serotypes in a single reaction.

### 2.5. Next-Generation Sequencing and Data Processing

The aforementioned PCR products were subjected to next-generation sequencing (NGS) using the Illumina platform (Biomic, Beijing, China) for whole-genome sequencing. Sample library construction was performed using the Illumina Nextera XT DNA Library Prep Kit (Illumina, San Diego, CA, USA), followed by paired-end sequencing on the Illumina NovaSeq 6000 platform (Illumina, San Diego, CA, USA). The initial raw data obtained were quality-controlled using *FastQC* (Babraham Bioinformatics, Cambridge, UK) to ensure sequence accuracy and completeness. Low-quality sequences were removed using *Trimmomatic* to ensure the reliability of subsequent analyses. The high-quality data generated post-sequencing were assembled into a draft genome using *SPAdes*. The quality of the assembly was evaluated based on the following metrics: Mean sequencing depth: 100×; Genome coverage: average 98.7%; N50 value: average 213 kb; Number of contigs: average 63; GC content: ~40.5%, consistent with the reference genome. All assembly results were submitted to the NCBI GenBank database. The high sequencing depth and coverage indicated good representativeness and reliability of the sequencing data, and the high N50 value suggested good assembly continuity, supporting the accuracy of subsequent analyses of resistance genes, virulence genes, and phylogenetics.

To investigate the epidemiological differences in *M. haemolytica* strains from different sources—comparing isolates from northwestern China with those from countries with developed livestock industries (e.g., Germany, the United States, and Canada)—nine isolates obtained in this study were analyzed alongside 30 complete genomes publicly available from the National Center for Biotechnology Information (NCBI), with 10 high-quality samples selected from each country. Genome annotation for all 39 strains was performed using Prokka (v1.14.6, Victorian Bioinformatics Consortium, Melbourne, Australia) to identify and label coding sequences (CDS), ribosomal RNA (rRNA), and transfer RNA (tRNA) genes [23] (Table S1). Annotation results were further analyzed and summarized. In addition, plasmids present in the genomes were identified using PlasmidFinder (v2.1.6) with default parameters.

### 2.6. Comparative Genomic Analysis

Virulence factor sequences were downloaded from the Virulence Factors of Pathogenic Bacteria database (VFDB; https://www.mgc.ac.cn/VFs/ (accessed on 2 April 2025)), and BLASTp analysis was performed using the NCBI BLAST tool (version 2.17.0) with an E-value threshold of 1 × 10^−5^. Hits with ≥90% coverage and >50% identity were retained as candidate virulence genes. A systematic comparison of the detected profiles across isolates was conducted to assess geographic variations in virulence gene composition (Table S2). Notably, several important *M. haemolytica* virulence determinants—such as *LktA*, *TonB*, *SodA*, *OmpA* and *FhaB* —were absent from VFDB and therefore were not detected through the initial approach. To address this, additional sequences of these key genes were retrieved from publicly available genomes and the relevant literature and subjected to BLASTp searches against our dataset. This supplementary strategy ensured a comprehensive annotation, capturing both VFDB-listed factors and species-specific virulence genes, thereby enhancing insights into the pathogenic potential of regional strain variations.

The reference genome of *M. haemolytica* (accession number: GCF_002285575.1) was downloaded from NCBI. Genomic sequences of 39 *M. haemolytica* strains were aligned to the reference genome using the ctgs parameter of Snippy (v4.6.0, https://github.com/tseemann/snippy (accessed on 2 April 2025)), followed by core genome single-nucleotide polymorphism (cgSNP) analysis. Recombinant regions were removed using Gubbins (v3.4), and a maximum likelihood phylogenetic tree was constructed with IQ-TREE (v2.4.0) [24].

Multilocus sequence typing (MLST) was performed on the *M. haemolytica* genomic data using MLST (v2.23.0). The typing results were organized, and a minimum spanning tree was generated using GrapeTree (v2.1) [25] (Table S3).

### 2.7. Drug Resistance Analysis

A detailed analysis of antibiotic resistance genes in the isolates was conducted using NGS data. First, the ResFinder database (Technical University of Denmark, Lyngby, Denmark) was utilized to annotate the antibiotic resistance genes identified in the sequencing results, enabling the recognition of genes associated with common antibiotic resistances [26]. Coupled with the Kirby-Bauer disk diffusion methodology (Thermo Fisher Scientific, Waltham, MA, USA), the susceptibility of each isolate to various antibiotics—including ciprofloxacin, azithromycin, gentamicin, levofloxacin, tylosin, and enrofloxacin (all antibiotics sourced from Sigma-Aldrich, St. Louis, MO, USA)—was tested to obtain phenotypic resistance data. Subsequently, a correlation analysis was performed between the identified resistance genes from the sequencing results and the phenotypic data from the antibiotic susceptibility tests, assessing the relationship between genotype and phenotypic resistance, thereby exploring the correlation between genetic resistance mechanisms and actual drug responses.

### 2.8. Pathogenicity Experiment

To evaluate the pathogenicity of the isolated strains, a mouse model was employed [27]. All animal procedures were approved by the Northwest A&F University Institutional Animal Care and Use Committee (No. XN2023-0619). A total of 30 healthy BALB/c mice (DaShuo, Chengdu, China) were selected, and each mouse was inoculated via intraperitoneal injection with the isolated *M. haemolytica* strains at a dose of 1 × 10^8^ CFU in 0.1 mL. The mice were grouped according to the geographic origin of the strains, including Shaanxi, Gansu, and Ningxia, with 10 mice in each group. Clinical symptoms in the mice were closely monitored within 24 h, including body temperature, respiratory distress, and reduced activity, and the mortality rate was recorded.

After the infection period, all mice underwent systematic necropsy, with a focus on collecting key tissue samples, including lung, liver, and spleen. These samples were fixed in 10% neutral formalin (Thermo Fisher Scientific, USA), followed by dehydration, paraffin embedding, and the preparation of 5 μm thick tissue sections. The sections were stained with hematoxylin and eosin (H&E) (Sigma-Aldrich, USA) and examined under an optical microscope (Olympus, Japan) to observe pathological features in the organs, with particular attention to inflammatory responses (such as infiltration of inflammatory cells), cellular necrosis, tissue congestion, and edema.

Using the virulence gene information obtained from NGS, quantitative PCR (qPCR) was further employed to detect the expression levels of target virulence genes. First, RNA was extracted from infected tissues, and a reverse transcription kit (Thermo Fisher Scientific, USA) was used to convert RNA into cDNA. Specific primers targeting the virulence genes were designed, and qPCR amplification was conducted using the SYBR Green methodology (Thermo Fisher Scientific, USA) on a fluorescence quantitative PCR instrument to assess the expression levels of each gene. Reference gene: *rpoB* was used as the reference gene, and its stable expression across groups was validated by GeNorm and NormFinder (M < 0.5, V < 0.15); the relative expression levels were calculated using the 2^−ΔΔCt^ method; three technical replicates for each sample. The expression data, after log_2_ transformation, were used for subsequent statistical analysis, and the linear relationship between virulence gene expression levels and pathogenicity indicators was assessed using Pearson correlation coefficients.

### 2.9. Data Analysis

The statistical analysis of the experimental data was completed using *SPSS 20* and R software(version 4.4.1). To assess the differences in the isolation rates of *M. haemolytica* among samples from different regions, the chi-square test (χ^2^ test) or Fisher’s exact test was used for intergroup comparisons. For the analysis of continuous variables such as resistance gene carriage and virulence gene expression levels, the Shapiro–Wilk normality test and Levene’s test for equality of variances were first conducted. If the data met the assumptions of normal distribution and equality of variances, a two-way ANOVA was used to evaluate the effects of geographical origin and gene type on expression levels and to test the interaction between the two factors; if the assumptions were not met, the non-parametric Kruskal–Wallis H test was used for multiple-group comparisons, followed by Dunn’s post hoc test for pairwise comparisons. All multiple comparisons were corrected using the Bonferroni correction to control the family-wise error rate. A difference was considered statistically significant only when the corrected *p*-value was less than 0.0033. To explore the potential associations between virulence gene expression and resistance phenotypes, as well as the correlation between virulence gene distribution and strain pathogenicity, Pearson correlation analysis was performed to evaluate the linear relationship between the two variables. A significance level of *p* < 0.05 was considered statistically significant.

## 3. Results

### 3.1. Distribution of Isolated Bacterial Strains

In this study, a total of 27 lung tissue samples from infected sheep were collected, from three northwest provinces of China (Gansu, Ningxia, Shaanxi). After culture, biochemical tests, and 16S rRNA gene sequencing identification, 9 strains of *M. haemolytica* were isolated, achieving an isolation rate of 33.33%. Specifically, the isolation rate in Shaanxi (6/9, 66.67%; 95% confidence interval [CI]: 36.1–97.2%) was significantly higher than those in Gansu (2/9, 22.22%; 95% CI: 0.0–49.0%) and Ningxia (1/9, 11.11%; 95% CI: 0.0–31.9%). The serotypes of the nine *M. haemolytica* isolates were determined using a validated multiplex PCR assay. The results showed that 33.33% (3/9) of isolates were identified as serotype A1, 55.56% (5/9) as serotype A2, and 11.11% (1/9) as serotype A6 (Table 2). Geographic distribution analysis revealed that A2 strains were predominantly found in Shaanxi Province, whereas the sole A6 strain was isolated from Gansu Province.

### 3.2. Virulence Gene Comparison Analysis

**Figure 1:**
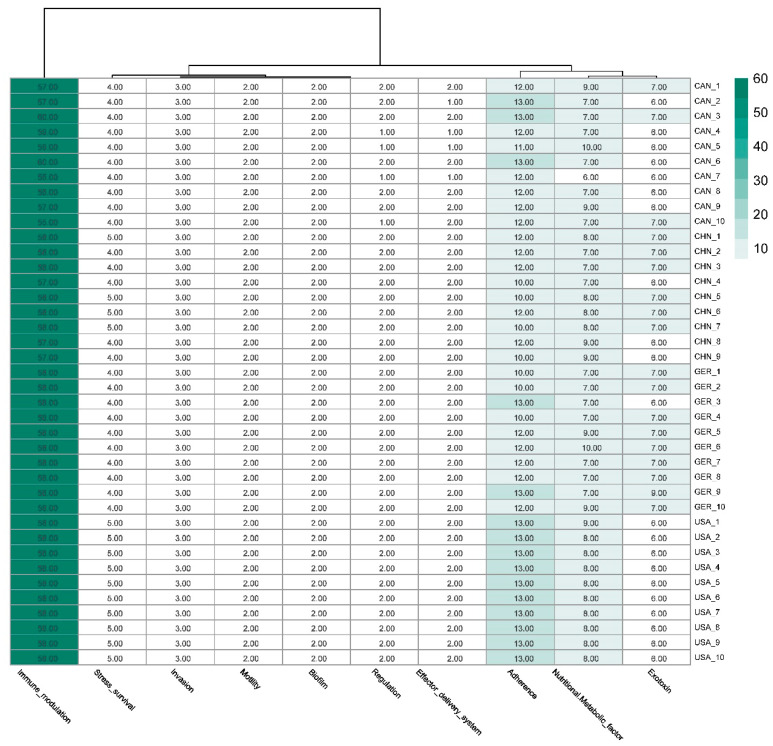
Distribution of virulence factors. The x-axis represents the types of virulence factors, and the y-axis includes the IDs of the 39 strains. Virulence factors were categorized into 10 distinct functional groups as illustrated in the figure. Among these, immune modulation represents the largest functional category, with an average of 57.7 genes per strain. This indicates that *M. haemolytica* predominantly adopts immune modulation strategies during pathogenesis, establishing persistent infections by interfering with both the recognition and effector mechanisms of the host immune system.

NGS and BLASTp analyses revealed a substantial repertoire of virulence-associated genes (n = 2931) across 39 *M. haemolytica* strains from China, North America, and Europe, spanning 102 distinct protein types (Figure 1).

Among specific virulence genes identified, *lktA* (hemolysin) was universally present (100%), followed by *fhaB* (filamentous hemagglutinin adhesin) at 97.44%, *sodA* (superoxide dismutase), *hly* (hemolysin), and *omp* (outer membrane protein), all with detection rates above 90%. In contrast, *tonB* (energy transport protein) was less prevalent, appearing in 79.49% of strains.

**Figure 2:**
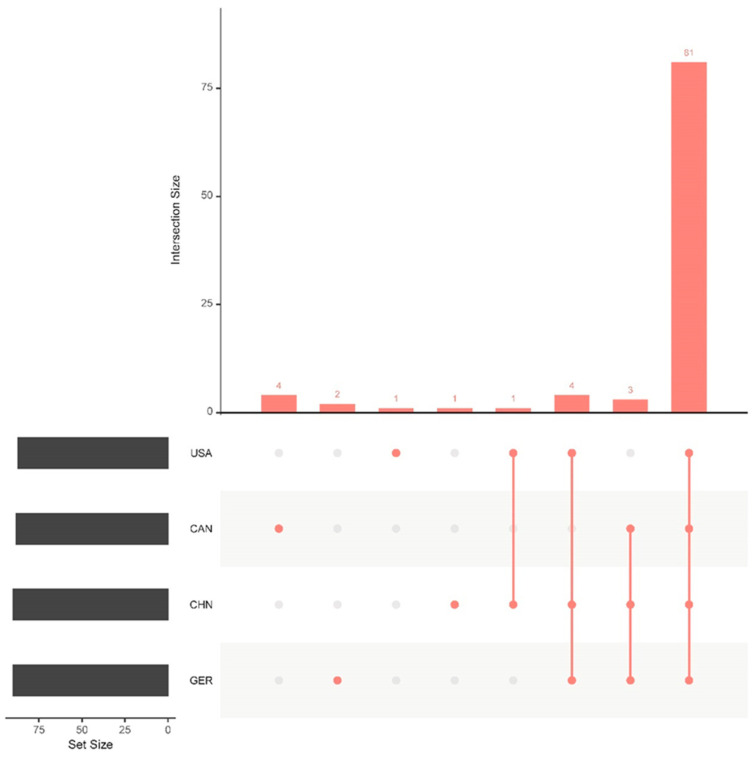
Distribution of virulence factors among different strains. The x-axis represents the intersection relationships of the four strains, and the y-axis includes the IDs of the 39 strains. The bar chart on the left shows the number of types of virulence factors present in each of the four groups.

These factors play diverse roles—including secretion of extracellular enzymes and toxins, synthesis of lipopolysaccharides and capsules, iron acquisition and metabolism, membrane transport, and metabolic regulation—with most being conserved across strains from all regions (Figure 2). Together, these findings indicate a robust core virulence repertoire within *M. haemolytica*, with both common and variable elements across geographical populations, reflecting their potential roles in pathogenicity.

### 3.3. cgSNP and Phylogenetic Analysis of Genomes

**Figure 3:**
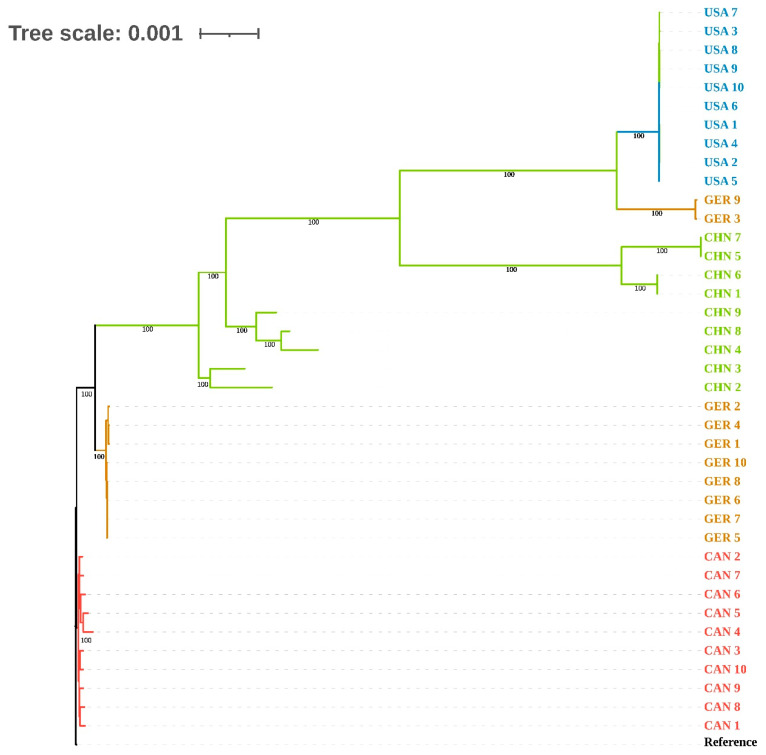
Phylogenetic relationship of 39 strains. “Reference” represents the reference genome GCF_002285575.1, and different colors indicate the geographical origin of the strains.

A total of 21,940 SNP sites were identified in this study. Phylogenetic tree analysis was performed based on these SNP sites. The results showed that, except for the CHN group, the strains from the USA, GER, and CAN groups exhibited very short branch lengths on the phylogenetic tree, indicating that the genetic differences between these strains were relatively small, suggesting they may originate from a recent common ancestor or have undergone similar evolutionary paths. However, it is noteworthy that the GER 9 and GER 3 strains from the GER group clustered with strains from the USA group, suggesting that GER 9 and GER 3 may not be of local origin, but rather represent foreign strains (Figure 3). This clustering pattern may reflect the occurrence of inter-regional transmission events during the evolutionary process or the possibility that these strains originated from a widely distributed common ancestor. Additionally, the strains in the CHN group showed longer branch distances on the phylogenetic tree, indicating greater genetic diversity within this group. This could be closely related to the unique local ecological environment, host species, or other ecological factors, leading to a more complex evolutionary pattern.

### 3.4. Genomic MLST Analysis

**Figure 4:**
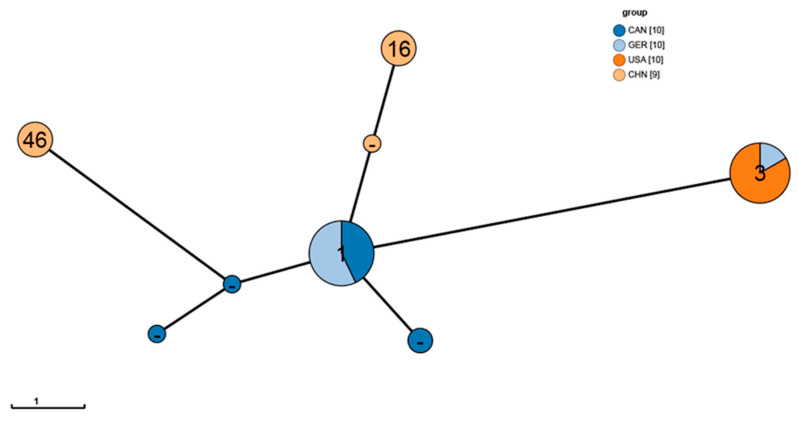
Minimum spanning tree of MLST for the 39 strains. Each circle represents an ST, with the size of the circle reflecting the frequency of each ST in the dataset. The number of different alleles is indicated by the ST, and the lines connecting the circles represent genetic distances with level values indicated on the labels. ST3 and ST4 cluster at the same node, indicating a relatively close genetic distance between them.

The MLST analysis results showed that 28 strains were classified into five known sequence types (STs), specifically ST 1 (8 strains), ST 3 (10 strains), ST 4 (2 strains), ST 16 (4 strains), and ST 46 (4 strains). Additionally, 11 strains could not be assigned to any known ST and were labeled as unknown STs (Figure 4). The analysis revealed that strains from the same geographic origin often share the same ST, suggesting that geographical origin influences the genetic differentiation of the strains to some extent. For example, all strains from the USA belonged to ST 3; strains from China (CHN) were primarily classified as ST 16 and ST 46; while strains with unknown ST mainly originated from Canada (CAN). Among the strains from Germany (GER), except for GER 3 and GER 9, the rest of the strains were of ST 1 type, while GER 3 and GER 9 belonged to ST 4. Furthermore, in the minimum spanning tree, GER 3 and GER 9 clustered with the USA strains of ST 4 at the same node. This phenomenon suggests that GER 3 and GER 9 might have an evolutionary history similar to the USA strains or may have undergone cross-regional transmission, which corroborates the phylogenetic results.

### 3.5. Drug Resistance Analysis

#### 3.5.1. Distribution of Drug Resistance and Related Genes

For the 9 strains of *M. haemolytica*, the sensitivity to six antibiotics was tested using the Kirby-Bauer disk diffusion methodology. The results indicated that *M. haemolytica* strains exhibited markedly higher sensitivity to ciprofloxacin, azithromycin, gentamicin, and levofloxacin versus tiamulin and enrofloxacin, for which resistance was markedly higher than that observed for the other antibiotics. Further analysis of antibiotic resistance genes in the 9 isolates was conducted using NGS technology. The ResFinder database was utilized to identify genes associated with antibiotic resistance, primarily including quinolone resistance gene (*qnr*), erythromycin resistance methylase B gene (*ermB*), aminoglycoside acetyltransferase (6′-N-acetyltransferase) Ib gene (*aac(6′)-Ib*), antibiotic class A transporter gene (*vgaA*), and DNA gyrase A subunit gene (*gyrA*) (Table 3).

#### 3.5.2. Association Analysis Between Genotype and Phenotype Drug Resistance

**Figure 5:**
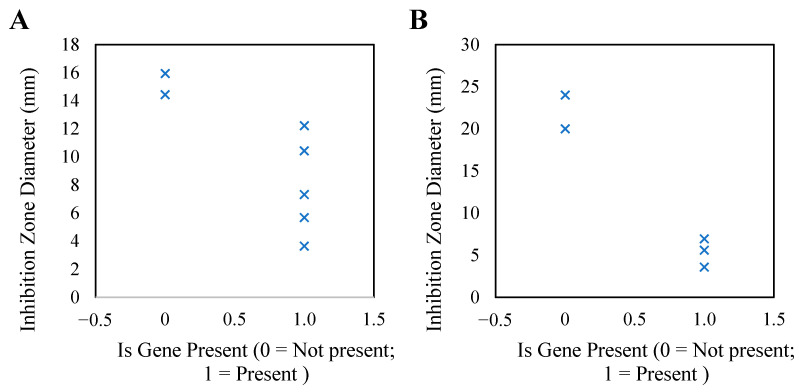
Association between tiamulin and enrofloxacin resistance genotypes and phenotypes ((**A**): *vgaA* gene; (**B**): *gyrA* gene). (**A**): The diameters of the inhibition zones of strains carrying the *vgaA* gene were generally small, but a few non-carriers also exhibited low sensitivity, suggesting that they might achieve resistance through other mechanisms. (**B**): All *gyrA*-positive strains were found to be clearly resistant, but some negative strains still showed moderate resistance, indicating that this gene is not the sole determinant.

Statistical analysis revealed that among the 7 strains of *M. haemolytica* resistant to tiamulin, 5 strains (71.43%) carried the *vgaA* gene, whereas among the 5 strains resistant to enrofloxacin, 3 strains (60%) carried the *gyrA* gene. By correlating the resistance gene data from NGS (presence of resistance genes coded as 0 = absent, 1 = present) with the phenotypic data from antibiotic sensitivity assays (measured as inhibition zone diameter in mm), we investigated the relationship between specific genotypes and phenotypic resistance (Figure 5). The results indicated that strains carrying resistance genes (*X* = 1) typically displayed smaller inhibition zone diameters, suggesting a stronger resistance to antibiotics. Notably, a few outlier strains lacking the expected gene still displayed reduced zones, suggesting that additional resistance mechanisms (such as efflux pumps or compensatory mutations) may also play a role.

### 3.6. Pathogenicity Analysis

By observing the changes in body temperature, respiration, activity, and mortality of 30 mice over 24 h (Table 4), the study found that all mice exhibited significant pathological symptoms, with 24 mice (80%) succumbing to the infection. In the Shaanxi group, all mice displayed symptoms, and all died; in the Gansu group, all mice showed symptoms, with 8 (80%) dying; in the Ningxia group, all mice exhibited symptoms, with 6 (60%) deaths. Notably, the Shaanxi group exhibited higher levels of pathological manifestations and mortality, suggesting marked differences in the pathological responses and mortality rates among mice infected with strains from different regions, which may be closely associated with variations in virulence genes among these strains.

Based on the data in Table 3, further calculations were made regarding the occurrence of pathological symptoms such as elevated body temperature, labored breathing, and reduced activity in the mice. The results shown that the proportion of mice exhibiting elevated body temperature (25/30 83.33%) and labored breathing (29/30 96.67%) was notably higher than that of those with reduced activity (17/30 56.67%), indicating that the clinical manifestations caused by the strains isolated in this study were primarily characterized by elevated body temperature and respiratory abnormalities, with reduced activity being less common. This phenomenon suggests that the pathogen causes rapid onset of disease in the mice, with the primary initial symptoms concentrated on systemic inflammatory responses and respiratory system damage. This may be closely related to the effect of bacterial toxins (such as the lktA hemolysin) on vascular permeability and lung tissue. These results provide important reference for future pathological mechanism studies and clinical symptom monitoring.

### 3.7. Pathological Observation Results

**Figure 6:**
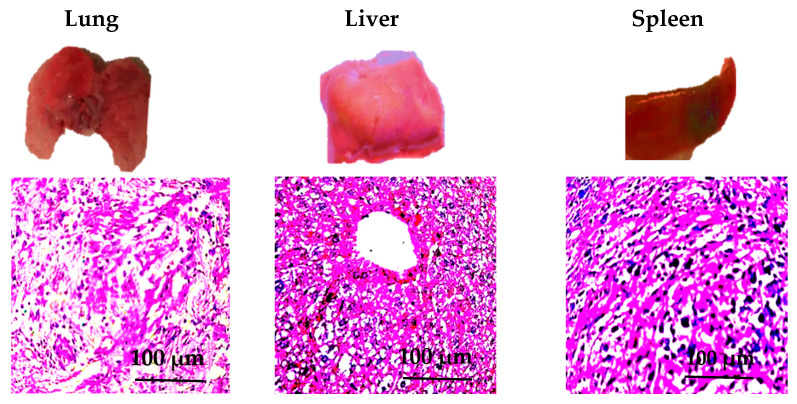
Tissue samples of the lung, liver, and spleen with H&E staining results. **Lung**: Gross inspection reveals congestion and enlargement; under the microscope, thickening of the alveolar septa, infiltration of inflammatory cells, and accumulation of exudate are observed, indicating acute inflammatory response and impaired lung function. **Liver**: Gross inspection reveals congestion and edema; under the microscope, aggregation of inflammatory cells around the central vein and hepatocyte necrosis are observed, indicating hepatic parenchymal injury. **Spleen**: Gross inspection reveals enlargement with white necrotic foci; under the microscope, widespread infiltration of inflammatory cells in the splenic pulp and disordered structure are observed, reflecting activation of the immune system and tissue damage.

The study conducted systematic pathological observation on all mice, focusing on key organs such as the lungs, liver, and spleen. The lungs exhibited congestion and enlargement, with observable thickening of the alveolar septa. A large influx of inflammatory cells infiltrated the lung tissue, and some areas showed evidence of cell necrosis. Exudate was present within the alveoli, resulting in impaired lung function (Figure 6-Lung).

The liver tissue showed signs of congestion and edema, with localized areas of hepatocyte necrosis. Clusters of inflammatory cells were observed, particularly around the central veins, indicating an inflammatory response. The structural integrity of the liver was compromised, displaying pronounced pathological changes (Figure 6-Liver).

The spleen was enlarged and presented with noticeable areas of white necrosis, particularly in the splenic pulp region. Observable infiltration of inflammatory cells within the spleen was noted, and the structure was damaged, reflecting an immune response to the infection (Figure 6-Spleen).

### 3.8. Correlation Analysis Between the Expression Levels of Virulence Genes and Pathogenicity

To further investigate the actual expression levels of virulence genes and their impact on pathogenicity, qPCR analysis was conducted on the virulence genes lktA, tonB, and sodA, which were frequently detected in the NGS data, in both the infected and control groups. The relative expression levels of the lktA, tonB, and sodA genes in the infected group were 8.1 ± 0.5, 4.5 ± 0.4, and 3.2 ± 0.3, respectively, while in the control group, the values were 1.0 ± 0.2, 1.2 ± 0.3, and 1.1 ± 0.2 (Table 5). The expression levels of lktA, tonB, and sodA in the infected group were drastically higher than those in the control group (*p* < 0.05), with the most notable upregulation observed for the lktA gene, which increased approximately eight-fold.

To investigate the correlation between the expression levels of virulence genes and the pathogenicity of *M. haemolytica*, a comprehensive analysis integrating the qPCR results, clinical symptoms, pathological changes, and mouse mortality rates was conducted. Additionally, a multiple linear regression model was employed to further validate the impact of these factors (Table 6).

In terms of clinical symptoms, the high expression of the *lktA* gene was highly correlated with severe clinical symptoms such as dyspnea and reduced activity (*r* = 0.78, *p* < 0.01). The *tonB* gene exhibited a moderate correlation with elevated body temperature (*r* = 0.60, *p* < 0.05), while the expression of the *sodA* gene showed a positive correlation with the infiltration of inflammatory cells (*r* = 0.65, *p* < 0.05).

Regarding pathological changes, the expression of the *lktA* gene was strongly correlated with tissue necrosis and inflammatory infiltration in the lungs (*r* = 0.82, *p* < 0.01). The *tonB* gene showed a moderate correlation with congestion and edema in the liver and spleen (*r* = 0.68, *p* < 0.05), while the *sodA* gene was associated with oxidative stress responses in the lungs (*r* = 0.63, *p* < 0.05).

As for mortality rates, high expression of the *lktA* gene was significantly positively correlated with mouse mortality (*r* = 0.85, *p* < 0.01), while *tonB* and *sodA* genes exhibited moderate to weak correlations with mortality (*r* = 0.55 and *r* = 0.50, *p* < 0.05, respectively).

Multiple linear regression analysis indicated that the *lktA* gene explained the highest proportion of pathogenic variation at 65% (*p* < 0.01), suggesting it is a major pathogenic gene. In contrast, *tonB* and *sodA* accounted for 30% and 25% of the pathogenic variation, respectively (*p* < 0.05), less pronounced than that of the *lktA* gene.

## 4. Discussion

This study systematically analyzed the distribution of *M. haemolytica* isolates from sheep in the three northwestern provinces of China, along with their serotype characteristics, the distribution of virulence and antibiotic resistance genes, and the relationship between these factors and pathogenicity. These findings provide significant insights into the epidemiology and pathogenic mechanisms of *M. haemolytica*.

In this study, 9 isolates of *M. haemolytica* were obtained from 27 lung tissue samples collected from diseased sheep in the three northwestern provinces of China, achieving a separation rate of 33.33%. Most recent studies report relatively low isolation rates of *M. haemolytica*; for example, one investigation in cattle in China recorded an isolation rate of only 2.44%, and other global studies in ruminants reported rates of 7.2% and 18.82% [28,29,30]. Although the sample size in our work is modest, the markedly higher isolation rate observed in our study underscores the significance and novelty of our findings. Notably, the isolation rate in Shaanxi was significantly higher than in other regions, likely due to the unique climatic conditions and grazing practices in the province, which may render local sheep populations more susceptible to *M. haemolytica* infection. Moreover, the increasing prevalence of intensive, high-density sheep rearing—whether through indoor housing or tightly packed grazing—probably amplifies this vulnerability by increasing pathogen exposure, limiting ventilation, and elevating stress-induced immune suppression [31]. These combined climatic, husbandry, and management pressures likely explain the elevated isolation rate in Shaanxi, and they suggest that targeted interventions—improving ventilation, reducing stocking density, optimizing shelter design, and adjusting grazing schedules—could effectively reduce disease burden [32].

Serotyping revealed that all 9 isolates of *M. haemolytica* belonged to serotypes A1, A2 and A6, with A2 strains constituting 55.56% of the total. Previous studies indicated that A2 strains are commonly associated with severe pneumonia and septicemia, whereas A6 strains exhibit relatively lower pathogenicity during infections [33,34]. The prevalence of A2 strains is higher in Shaanxi, this geographical distribution indicates their potential strong adaptability and transmissibility, highlighting the necessity to enhance vaccination and control measures targeting A2 strains in high-infection areas such as Shaanxi.

Integrating sequences from different countries enables us to better understand the evolutionary trajectories and potential dissemination pathways of *M. haemolytica*. Through genomic sequencing and BLAST analysis, multiple virulence genes in *M. haemolytica* were identified, with the *lktA* gene showing the highest detection rate (100%) among the 9 strains. This indicated that the *lktA* hemolysin plays a crucial role in the pathogenic mechanism of this species. This finding is consistent with existing literature that highlights *lktA* as a significant virulence factor in *M. haemolytica* [35,36]. Further qPCR analysis revealed that the expression levels of the *lktA*, *tonB*, and *sodA* genes in the infection group were considerably higher than those in the control group (*p* < 0.05), with the most pronounced upregulation observed for the *lktA* gene, reaching approximately eightfold. Multiple linear regression analysis indicated a notable positive correlation between the high expression of *lktA* and severe clinical symptoms, pathological changes, and elevated mortality rates, positioning it as a critical determinant of the strain’s pathogenicity. Although *tonB* and *sodA* were also associated with pathogenicity, their effects were relatively weaker. This finding aligns with other research outcomes, emphasizing the central pathogenic role of *lktA* in *M. haemolytica* infections [37].

The antibiotic susceptibility testing results indicated that *M. haemolytica* exhibited high sensitivity to ciprofloxacin, azithromycin, gentamicin, and levofloxacin, while there was a significant increase in resistance to tiamulin and enrofloxacin, particularly with a resistance rate for tiamulin reaching 77.78%. Genetic analysis using the ResFinder database showed that high resistance to tiamulin and enrofloxacin, along with the detection rates of their respective resistance genes *vgaA* and *gyrA*, increased to 66.67%. The *vgaA* gene encodes an ABC-F family ribosome-protection protein that binds directly to the 50S ribosomal subunit and displaces antibiotics—such as pleuromutilins, streptogramin A and lincosamides—from the peptidyl-transferase center, thereby preserving translation in the presence of these drugs [38,39]. In contrast, *gyrA* encodes the A-subunit of DNA gyrase, the primary fluoroquinolone target in Gram-negative bacteria; resistance arises via point mutations within the quinolone-resistance-determining region (QRDR) of GyrA, which reduce drug binding and facilitate higher-level resistance through additional mechanisms such as increased efflux or porin modifications [40,41]. In addition, although the MexAB-OprM efflux-pump system is well characterized in *Pseudomonas aeruginosa*, it has not been clearly demonstrated in *M. haemolytica* [42]. In Gram-negative bacteria generally, this gene family is widespread and highly conserved. While ResFinder can identify some efflux-pump genes (e.g., acrAB), its capacity to detect species-specific or novel efflux systems remains limited [43]. Because no functional validation was performed—such as using efflux inhibitors (e.g., Carbonyl cyanide m-chlorophenylhydrazone, CCCP), RT-qPCR expression analysis or knockout assays—the role of efflux pumps in our isolates cannot be confirmed. Moreover, this study focused exclusively on *gyrA* sequence changes and did not systematically examine other topoisomerase-related targets (e.g., *parC*, *grlA*), so the possibility of multi-target mutation contributions cannot be excluded. The limited update frequency of ResFinder further restricts its coverage of newly emerging or species-specific resistance determinants. In the few strains that displayed phenotypic resistance without identifiable known genes, possible explanations include: (1) novel resistance genes absent from the database; (2) low-copy or chimeric elements reducing detection sensitivity; or (3) unannotated plasmid-mediated resistance genes. Future work should address these gaps via plasmid extraction and sequencing, conjugation studies to assess gene mobility, as well as Sanger sequencing verification of key loci and functional assays (e.g., gene knockout or heterologous expression). It is well established that horizontal gene transfer (HGT)—via conjugation, transformation or transduction—facilitates rapid acquisition of resistance genes and mobile genetic elements (MGEs) across bacterial populations [44,45]. Recombination further enhances bacterial adaptability by integrating foreign DNA fragments into recipient genomes, thereby accelerating the dissemination of multi-drug resistance traits [46]. In the context of ovine-derived *M. haemolytica*, such processes may account for the elevated prevalence of *vgaA*/*gyrA* genes and the regional clustering patterns detected in this study, underscoring the need for future investigations into plasmids, integrative conjugative elements and other mobile platforms. Collectively, these findings indicate that antimicrobial resistance in *M. haemolytica* is largely driven by genetic determinants such as *vgaA* and *gyrA* [47,48]. Additionally, this study found that strains carrying resistance genes exhibited smaller inhibition zone diameters, further confirming the significant association between genotype and phenotypic resistance. These findings remind us that, in regions with elevated resistance, it is crucial to strengthen the regulation of antibiotic usage—particularly to prevent the overuse of tiamulin and enrofloxacin—and thereby mitigate the risk of disseminating resistant strains.

In the pathogenicity experiments conducted on mice, isolates from different regions exhibited varied pathogenicity, with strains from Shaanxi demonstrating the highest virulence. Pathological observations revealed significant lesions in target organs, including the lungs, liver, and spleen of infected mice, primarily characterized by pulmonary necrosis, hepatic congestion, and white necrotic foci in the spleen. These lesions were closely associated with the high expression of virulence genes, particularly the elevated expression of *lktA*. Such regional divergence in virulence may reflect compounded ecological and management pressures: elevated stocking densities, increased inter-flock animal movements and heightened environmental stress can amplify pathogen transmission and intensify host susceptibility. Indeed, the dynamics of *M. haemolytica* transmission have been shown to accelerate significantly under close-contact conditions in densely populated flocks [49,50]. These combined factors likely contributed to the enhanced virulence observed in Shaanxi isolates and highlight the value of integrating detailed meteorological, husbandry and animal-movement metadata in future virulence investigations.

Although only 27 lung-tissue samples were collected and 9 *M. haemolytica* isolates obtained, this reflects the inherent logistical and ethical constraints associated with field sampling in sheep flocks of northwest China. In typical production settings, sheep with respiratory symptoms are rapidly isolated and treated empirically—substantially reducing the opportunity to recover untreated, pathogen-positive specimens. Moreover, for analytic specificity, only lung tissues from sheep that died solely of respiratory disease (i.e., excluding concurrent conditions such as lymphadenitis, mastitis or foot-rot) were included. Although this strict screening reduced the sample size, it improved the internal validity of our conclusions. A murine infection model was employed for comparative virulence assessment; despite known immunological and physiological differences between mice and sheep, laboratory mice offer a reproducible and ethically acceptable platform for initial pathogenicity screening. Indeed, multiple studies have employed murine models to investigate *M. haemolytica* pathobiology. For example, mice deficient in interferon-inducible protein (IFI204) exhibited markedly increased bacterial loads, severe lung pathology, and reduced survival upon challenge, highlighting the critical role of inflammasome signaling in host defense [51]. Other work using RNA-seq in infected mice revealed key chemokine pathways and immune-regulatory mechanisms in splenic tissue during *M. haemolytica* challenge [27]. Additionally, a recombinant chimeric vaccine combining leukotoxin and outer membrane protein elicited strong bactericidal and neutralizing responses in mice [52]. Together, these findings support the use of the murine model as a robust and ethically manageable platform for dissecting *M. haemolytica* virulence, host immune interactions, and preliminary vaccine efficacy studies before moving on to ovine-specific challenge systems. Consequently, this investigation should be considered a foundational study that generates baseline data on the regional distribution, virulence, and antimicrobial resistance characteristics of ovine *M. haemolytica*. In our future work, we plan to broaden the framework of sample collection to include environmental isolates, clinically healthy carrier animals, and comprehensive metadata covering farm stocking densities, climatological variables and management practices. Additionally, we intend to establish a controlled ovine challenge model to validate the trends observed here and to delineate the ecological drivers of *M. haemolytica* epidemiology in small ruminant populations.

Compared to studies from other regions, this research revealed the prevalence and virulence gene characteristics of *M. haemolytica* in the three provinces of Northwest China. Similar studies conducted abroad have also shown regional variations in the prevalence of *M. haemolytica* and the distribution of major virulence genes, particularly in Europe and North America, where serotype A2 and the *lktA* gene are predominant [53,54]. The findings of this study provide important insights for further exploration of the pathogenic mechanisms of *M. haemolytica*, emphasizing the need for enhanced control measures tailored to the specific characteristics of strains in targeted regions to mitigate damage to livestock. Moreover, it is noteworthy that research on *M. haemolytica* has historically focused on bovine strains, with comparatively few studies addressing ovine-derived strains—despite evidence that sheep and goats also suffer significant illness and economic loss from this pathogen [55,56]. Our research focus on sheep-derived isolates thus addresses a crucial gap and underlines the need for increased attention to ovine *M. haemolytica* in future research, surveillance, and vaccine design.

## 5. Conclusions

This study presents the first comprehensive analysis of epidemiology, virulence-gene prevalence and their associations with pathogenicity and antimicrobial resistance in sheep-derived *M. haemolytica* isolates from three provinces in Northwest China. We observed prominent regional variation: isolates from Shaanxi displayed the highest virulence and resistance profiles. The identification of *lktA* as a major pathogenicity determinant and the elevated prevalence of key resistance genes signal an urgent need to strengthen antimicrobial stewardship in small-ruminant flocks. Given the predominance of serotypes A2 and A1 in our samples, our findings support the development of multivalent, region-specific vaccines. Moreover, the evidence emphasizes the importance of continuous genomic surveillance of circulating *M. haemolytica* lineages alongside antimicrobial-use monitoring. Finally, our work underscores the vital role of a One Health approach integrating veterinary, environmental and molecular surveillance to effectively control *M. haemolytica* infections in small-ruminant systems.

## Figures and Tables

**Table 1:** Serotype PCR identification primers.

Gene	Serotype	Sequence	DNA Fragment
*Hyp*	A1	F: 5′-CATTTCCTTAGGTTCAGC-3′	306 bp
		R: 5′-CAAGTCATCGTAATGCCT-3′	
*Core2*	A2	F: 5′-GGCATATCCTAAAGCCGT-3′	160 bp
		R: 5′-AGAATCCACTATTGGGCACC-3′	
*TupA*	A6	F: 5′-TGAGAATTTCGACAGCACT-3′	78 bp
		R: 5′-ACCTTGGCATATCGTACC-3′	

**Table 2:** Collection region, source, Serotype of the *M. haemolytica*.

Isolate	Region	Source	Serotype	Accession Number
MH1	Shaanxi	Ovine	A2	SAMN52441444
MH2	Gansu	Ovine	A6	SAMN52441445
MH3	Shaanxi	Ovine	A1	SAMN52441446
MH4	Shaanxi	Ovine	A1	SAMN52441447
MH5	Shaanxi	Ovine	A2	SAMN52441448
MH6	Shaanxi	Ovine	A2	SAMN52441449
MH7	Shaanxi	Ovine	A2	SAMN52441450
MH8	Ningxia	Ovine	A1	SAMN52441451
MH9	Gansu	Ovine	A2	SAMN52441452

**Table 3:** Sensitivity testing of 9 isolated strains to six antibiotics and results of antibiotic resistance gene detection.

Antibiotic	Sensitivity (%)	Intermediate (%)	Mean Inhibition Zone Diameter (mm) ± SD	Resistant (%)	Associated Resistance Gene
Ciprofloxacin	8 (88.89)	0 (0.00)	17.64 ± 1.25	1 (11.11)	qnr
Azithromycin	8 (88.89)	0 (0.00)	16.36 ± 1.83	1 (11.11)	ermB
Gentamicin	9 (100)	0 (0.00)	18.54 ± 1.02	0 (0.00)	aac(6′)-Ib
Levofloxacin	9 (100)	0 (0.00)	17.86 ± 1.12	0 (0.00)	qnrA
Tiamulin	1 (11.11)	1 (11.11)	8.25 ± 2.48	7 (77.78)	vgaA
Enrofloxacin	3 (33.33)	1 (11.11)	9.13 ± 2.72	5 (55.56)	gyrA

**Table 4:** Pathological manifestations and mortality statistics of mice in each group.

Group	Total	Number of Pathological Manifestations	Proportion (%)	Number of Deaths	Mortality Rate (%)	Elevated Body Temperature	Shortness of Breath	Reduced Activities
Shaanxi	10	10	100	10	100	9	10	8
Gansu	10	10	100	8	80	8	10	5
Ningxia	10	10	100	6	60	8	9	4
Total	30	30	100	24	80%	25	29	17

**Table 5:** Relative expression levels of various genes in the infected group and control group.

Gene	Infected Group (n = 24)	Control Group (n = 6)	Expression Fold Change	*p*
*lktA*	8.1 ± 0.5	1.0 ± 0.2	8.1	<0.01
*tonB*	4.5 ± 0.4	1.2 ± 0.3	4.5	<0.05
*sodA*	3.2 ± 0.3	1.1 ± 0.2	3.2	<0.05

**Table 6:** Correlation analysis between expression levels of virulence genes and various indicators of pathogenicity.

Gene	Clinical Symptom Correlation (r)	Correlation of Pathological Changes (r)	Mortality Correlation (r)	Pathogenicity Explanation Ratio (R2)	*p*
*lktA*	0.78 (Difficulty breathing and reduced activity)	0.82 (Pulmonary necrosis, inflammatory infiltration)	0.85	65%	<0.01
*tonB*	0.60 (Elevated body temperature)	0.68 (Liver congestion and edema)	0.55	30%	<0.05
*sodA*	0.65 (Inflammatory cell infiltration)	0.63 (Oxidative stress response)	0.5	25%	<0.05

## Data Availability

The authors confirm that all data is fully available without restriction. The sequencing reads were deposited into the NCBI database (Accession Number: PRJNA1338480).

## Supplementary Materials

The following supporting information can be downloaded at: https://www.mdpi.com/article/10.3390/ani15233492/s1, Table S1: Genome assembly and annotation summary of *Mannheimia haemolytica* isolates; Table S2: Virulence factor annotation results of *Mannheimia haemolytica* strain; Table S3: MLST allele profile of *Mannheimia haemolytica* isolates.
